# Neuroergonomics: Where the Cortex Hits the Concrete

**DOI:** 10.3389/fnhum.2019.00115

**Published:** 2019-04-12

**Authors:** P. A. Hancock

**Affiliations:** Department of Psychology, Institute for Simulation and Training, University of Central, Orlando, FL, United States

**Keywords:** neuroergonomics, adaptive systems, human-machine systems, automation, autonomy

## Introduction

This present article considers the nature and impact of the vital contributions of Raja Parasuraman and the way in which his brain-child–neuroergonomics–has begun to flourish and blossom in our contemporary world. By unlocking the secrets of brain activity and then finding mechanisms and technologies to elaborate such processes more directly into the world, he has provided us with a clear vision for the future of human-machine interaction. However, it always useful to start by consider antitheses. Thus, I begin my present exploration with the view from radical behaviorism, in some ways the opposite side of the coin from neuroergonomics. To behaviorist researchers, particularly if they are motivated by a utilitarian approach to the world, what happens in the brain may be considered of only passing, rather than vital, interest. This is not to say even the most ardent behaviorist denies the reality of cortical events; for that would be to misrepresent their position (see Watson, 1913; Chiesa, 1994). No, the simple fact is that for some such researchers, if you are able to predict, within a reasonable degree of tolerance, what behavioral sequelae derive from a particular set of environmental cues, then the knowledge of the intervening cortical activity can appear to be somewhat superfluous. For behaviorists, externality is all and associated brain states are of only diminished and diminishing concern.

And. in a somewhat analogous manner, this perspective on the (in)utility of an understanding of intervening (neurological) influences is of the same order of the proposal made by the philosopher Berkeley (1710) to support his idea of the non-existence of matter. Berkeley argued that the step of introducing matter as a mediating entity between the mind of God and the mind of man, was superfluous. This “additional,” and presumably unnecessary, step was even potentially insulting to a “perfect” God who would, presumably eschew inelegant and non-optimal strategies. Only a few now really take Berkeley's denial of matter seriously, and similarly few in our modern world would champion a pristine version of radical behaviorism. Certainly not Raja Parasuraman who saw great value in the potential for real-world applications from modern, emerging neuroscientific insights (Parasuraman, 2003). For most of us in the modern neurosciences then, however well the behaviorist's input-output specifications of the purported “black-box” work, like Pandora we still feel impelled to look inside. Berkeley's argument may however, come full circle. For surely a fully functional neuroergonomics will allow that the mind of one person be readily embedded in the perceived reality of all others. Thus, thesis and antithesis can always render some degree of synthesis.

In this brief article, I look at the genesis of Parasuraman's “neuroergonomic” vision, its growth to the present time, and some of the directions it might take in the near and more distant future. While the work evidently represents an homage to Parasuraman himself, it still raises critical points and debatable objections to neuroergonomics, most especially concerning the facile and untrammeled access (interaction) between the personal, private dimensions of thought and their unfiltered externalizations via modern-day, computer-mediated technology. While I do not find myself on the side of the ardent behaviorist with respect to any such philosophical “fence,” it is still reasonable to call for some limits and constraints upon the intimacy of the linkage between the cortex and the computer (and see Hancock, 2017).

## The Genesis of Neuroergonomics

While the term neuroergonomics was first coined by Parasuraman, the general idea of linking mind and machine is clearly not original to him (see e.g., Licklider, 1960). The growing degree of communication between humans and technology was inspired by some at the heart of the cognitive revolution.^1^ These included luminaries such as Craik (1947, 1948) and Broadbent (1958, 1971), in whose direct academic lineage Parasuraman can be found. In one important sense, Parasuraman's course was set when he entered doctoral work under the tutelage of Professor Roy Davies (Parasuraman and Davies, 1976). Between them, and perhaps with more emphasis here on Raja's contribution, they essentially resolved the confusion inherent in the then extant vigilance literature (see e.g., Buckner and McGrath, 1963). This resolution was accomplished through the implementation of the now rightly famous vigilance taxonomy (see Parasuraman, 1979; Davies and Parasuraman, 1982; Hancock, 2017). Yet, Parasuraman's grounding for developing neuroergonomics precedes even this doctoral phase of his career. For, his undergraduate work had been in electrical engineering (EE) and one can, if pushed, argue that neuroscience is one very specialized form of EE. Certainly, the formalizations and models of EE promote an affinity with the neurosciences in many of its forms.

However, beyond speculations on these associations, Raja's choice of research topic and subsequent exploration of vigilance served assuredly as his first formal stride toward the neuroergonomic conception. Clearly challenged by his advisor to clarify the vigilance concept (see e.g., Davies and Tune, 1969), this area of sustained attention has proven to epitomize the marriage of pure science and application in the real-world (see Mackworth, 1948, 1950; Hancock and Warm, 1989; Hancock, 2013). In point of fact, vigilance research emerged from a pure, real-world challenge and the attachment of the term “vigilance” was itself derived from Mackworth's search through then applicable neuroscientific conceptualizations of the time (see Head, 1923; Swash, 2008; see also the work of one of Head's colleagues and advisors, Rivers-Rivers, 1896). Slowly, and some might observe inevitably, Parasuraman's search for a theoretical explanation of the descriptive taxonomy he had distilled, embraced contemporary neuroscientific progress (Parasuraman, 1979). This development was facilitated by Raja's post-doctoral collaboration with Jackson “Jack” Beatty (see Parasuraman and Beatty, 1980). From a present-day perspective, the specific techniques they used, such as Electroencephalography (EEG) and eye movement assessment, might appear to be rather rudimentary, but in the middle to late 1970s they were at the very forefront of cortical exploration (and see e.g., Gath et al., 1983; Berka et al., 2007).

Parasuraman's investigations of advanced human-machine systems, which for him often meant complex aviation technologies (see e.g., Parasuraman et al., 1996; Parasuraman and Manzey, 2010), was accompanied by some very basic and fundamental contributions to the foundations of modern neuroscience (e.g., Parasuraman et al., 1992). Although his efforts in the practical and the theoretical domains can be linked to opportunistic and relevant funding successes, the desire for some form of hybrid sub-discipline was, for Parasuraman, more conceptually driven. Most especially, he became progressively more and more involved in human interaction with automation in which the essential “readiness of the operator to respond” is mediated not merely the success of collaborative interaction but dictated the very form which such interaction would be advised to take (and see Parasuraman and Mouloua, 1996). In part, this perspective perhaps under-estimates the purpose-directed work that he and his neuroscience colleagues conducted, on what might be termed pure “neuroscience inquiries.” Yet, these basic inquiries were at the heart of, and served to temper, the genesis of neuro-ergonomics (Parasuraman and Wilson, 2008).

Having been a longstanding colleague of Parasuraman (e.g., Hancock and Parasuraman, 1992, 2003) and having similar interests in general, and on several topics in particular (Hancock et al., 2000), it was quite natural that we discussed and wrote about his neuroergonomic conceptualization (Parasuraman and Hancock, 2004). It was a proposition that Raja discussed with and published on with several luminaries of our science (e.g., Kramer and Parasuraman, 2007). In contrast to the behavioristic proposition presented earlier, we both believed that a greater level of insight into the symphonic productions of the neural orchestra could provide exceptional opportunities to advance human-technology interaction (and see Hancock and Szalma, 2003). This whole enterprise m was then being facilitated by new and exciting brain assessment techniques (e.g., functional Near-Infrared Spectroscopy [fNIRS], and functional Magnetic Resonance Imaging [fMRI]) that opened up new empirical strategies to explain behaviors such as vigilance (Parasuraman, 1986) as well as providing neuroergonomic signals as input to advanced automation. For example, Parasuraman encouraged the fruitful explorations by Scerbo et al. in the creation of an EEG-based index of engagement for adaptive automation control (see e.g., Prinzel et al., 2000; Mikulka et al., 2002). It was in this way that my own original conceptualization of physiologically-mediated, adaptive systems (Hancock et al., 1985; Hancock and Chignell, 1987) could be enacted in the real world. Energized by the DARPA resources involved in the AugCog program, these human-machine forms of neuroergonomic adaptation are now becoming ever more ubiquitous. Such advances evidently involved many leaders in the area of human-machine interaction including critical contributions by those such as Sheridan and Wickens (see e.g., Parasuraman et al., 2000b) as well as those involved in the very first forms of adaptive human-machine systems such as Reising et al. (see Rouse, 1988; and Wilson and Russell, 2003a,b, 2007). But neuroergonomics remained Parasuraman's own.

## Within Pasteur's Quadrant: The Value of Use-Inspired Basic Research

Most certainly neuroergonomics is modulated by use-inspired research (Price and Behrens, 2003; Stokes, 2011). It does not stand apart from either pure neuroscience or applied ergonomics but brings the conceptual advances of neuroscience to the world. In this, it challenges the researchers in neuroscience with the practical demands of an ever more, technologically dominated world. Some would argue the use-inspired basic research program is a highly effective and workable model for virtually all of science (Sawyer and Hancock, 2018). Clearly, Parasuraman's conception resonated with many and, after his untimely death, motivated an International Conference first held in Paris in 2016 (and see publications derived therefrom; e.g., Ayaz and Dehais, 2019). A second meeting occurring in Philadelphia now presages the planning stages of its third, biennial meeting for 2020. Many have continued to explore ways in which insights from neuroscience can be enacted in the real-world including my own colleagues who explored how error-related negativity can be used as a pre-emptive signal for error prevention (Sawyer et al., 2017). Of course, we must set such investigations into the wider context of brain-machine interfaces that have been pursued in medicine and other realms, now joining with neuroergonomic developments. It is evident from the present volume concerning the enhancing of the linkage between brain and work, that the fecundity of the neuroergonomic conception lives on and promises to do so, at least for the foreseeable future. From the foregoing, it must be clear that I remain a strong advocate for neuroergonomics and thankful for Raja's fundamental and seminal identification; especially in the naming of the field. However, despite such an optimistic perspective and a vista of fruitful prognostications, neuroergonomics is not without its flaws and potential drawbacks. It is to these concerns that I turn next.

## The Dangers of Turning the Mind Inside-Out

The human narrative is essentially a highly and unwarrantedly, optimistic one. In both science and technology, the general zeitgeist is characterized and colored by a positive and enlightened, progressive attitude. Yet, as we are all aware, the developments of science, and their materialization in technology, can be used to pursue very different moral aims and practical goals (Hancock, 2009). Neuroergonomic developments are not exempt from this inherent ambivalence of utility. As I noted earlier, when speed is the primary imperative which is emphasized in a cybernetic, neuroergonomic assembly, then the propensity to speed up any intrinsic error can itself be even fatally facilitated. If neuroergonomics can be used to “short-circuit” some of the components of cognition, then unanticipated and most probably unwanted outcomes might well be facilitated also. For example, the perfection of neuroergonomic techniques might materialize thought into action, all in a moment. Here, a stray idea or passing fancy is then enacted, even if the individual regrets such thoughts almost he moment they have them (Yerdon et al., 2018; Matthews et al., 2019). Connections here to advanced weaponry will render the outcome of some such actions as irreversible. Here then, we need to countenance some limits upon neuroergonomic processing in the form of safety or reality checks. Such discussions certainly need to be had. Although we can assure and comfort ourselves that appropriate safeguards will be in place, such indemnifications are themselves not liable to work flawlessly.

Deception, or lie detection is one relevant example here of insufficiently specified and rather problematic neuroergonomic application. While there is current talk of enforcing mandatory lie detection to members of the current White House staff, the signal detection capacity of this technique is certainly not perfect. As Parasuraman realized Signal Detection Theory (SDT) techniques themselves are not perfectly enacted or reliable when applied to real-world circumstances (Parasuraman et al., 1997, 2000a). As a consequence, one would not wish to link many contemporary, operationally critical systems to the forms of elicited feedback in the classical types of lie detection investigations. Yet, as the range of methods and their diagnosticity and validity, both alone and in combination improve, so the capacity to enact accurate and neuro-cybernetic control itself matures. The issue of veridical detection is important beyond the specialized concerns of deception alone (and see Hancock, 2015). Like much of conscious thought, deception is a higher level function which is much more related to the content of ratiocination, as opposed to the processes that support it. The more general concern of the wider public may well be that neuroergonomics can “read your mind.” Thus, it is important to express the granularity of the interpretation of neuroergonomic signals. This will help affirm (pro tem), that the stimuli of concern for such input are much more allied to the actions of relatively small groups of neurons. It is, of course, true that neuroergonomics seeks to harness EEG signals as well as those from more recent measures (e.g., fNIRS). However, the principle goal, at the present time, is not to read the private contents of individuals' thought. This being said, science rolls on and the possibilities of misuse must be considered, as it must with all forms of automation and technology (Parasuraman and Riley, 1997). Perhaps, at this nascent stage, we might entertain a charter of those activities that neuroergonomics *should* and *should not* be used for. Yet I feel, that rather like Asimov's Three Laws of Robotics, these chartered actions might act more as pabulum then prohibition. I know such issues concerned Raja and should be respected and examined by the interdisciplinary area of science that he pioneered.

## A Brief Glimpse of the Future for Neuroergonomics

So what then of the future for neuroergonomics? We have to consider this in terms of both temporal horizon and contextual area. The temporal horizon can be conceived of as near-term events, likely within the next decade, and more extended projections, perhaps across the coming century. As for area context, we must look for developments in the neurosciences, the nature of work and general human activity and their fusion in the neuroergonomic enterprise. Across all of these time-scales and focus areas, the central leitmotif will, I think, be human-machine intimacy. At present, the connections between humans and their technology are slow, limited, and in reality rather absurdly constrained. We currently witness individuals in possession of hand-held super-computer linked to extensive networks and databases, hunting and pecking with fingers on ill-designed keyboard displays to enter language-mediated and language-limited instructions to achieve their purported goals. It has turned our contemporary world into unending lines of “*heads-down*,” oblivious persons, constantly and myopically attached to these limited portals (and see Hancock, 2016). Near-term technical developments will surely have to further expose and dissolve these highly limiting interface constraints. It is rather ludicrous that interactions with the most advanced of human-computational creations can even be countenanced as being limited by the quirky configuration of “QWERTY” keyboards. Although I do not wish to limit this point to any particular interface instantiation, I believe that significant progress can and will be made here at the interface. I see the line of evolution being characterized as “*the disappearance of the interface*.” Here, the physical contact between the user and their computational support system will not merely became small, faster, and more portable, but will begin to “evaporate,” as it were. In principle, any surface in a person's ambient environment can act as a “display” space (and see Hancock et al., 2015). Thus, personally “carrying” a display will, presumably and even shortly, be obviated altogether. I see this however, as closer to a century rather than a decade step of progress, although technologies like automated vehicles may well-serve to accelerate these, “not-on-body” option (Hancock et al., 2019a). Similarly, controls also will eventually be remotely enacted. The degree to which the physicality of such future human-computer interaction will be embedded in the uses, as opposed to being embedded in the world is simply a question of preferred (and currently most profitable) design options. Proximal and remote “intent inferencing,” and interfaces to support such intent inferencing will be crucial areas of technical and neuroergonomic development.

I have briefly mentioned the potential downsides of an untrammeled link between intention and action. In reality, we rather need now to be writing, if not laws, at least socially-agreed conventions on such limits, right at the present time. That we are not means that the future will continue to see *ad hoc* emergent patterns from an unplanned, and relatively “mindless” maw of realized technical innovations. Such unconstrained emergence portends potential disaster. For the neuroergonomic technologies, the battle of the coming decade remains “signal to noise” differentiation. We have an evident existence proof that patterns of activation in the brain do sum to produce intended actions in nominally “normal” human beings. But how to identify those patterns, how to model them, and how to predict them remain mountainous and prohibitive challenges. Yet many are climbing this mountain and work on the physical, prosthetic replication and replacement of such capacities for variously “disabled” individuals progresses apace. I am optimistic of punctate “successes” here, along this line of development in the near future. More understanding in this domain will also help us distinguish between simple, quantifiable processing “capacities,” and what the human brain actually achieves. I can well-countenance machine superiority in some closed domains, especially if floating point operations are used as the metric of capacity assessment (Moore, 1965; Kurzweil, 2005). What remains undecided is the relative capacities when we frame the two comparisons from a human perspective. The latter would feature measurements on dimensions such as trust, creativity, interest, etc. of which in artificial computational systems, we know almost nothing (Salmon et al., 2019). Yet, as neuroergonomics features, it is not human vs. computer, but rather a true emergent hybrid of the two which is the unit of interest. Once the interface barriers are down, we may have to re-cast the whole of psychology by asking questions like, what now is memory? and what is “selective attention” when all elements of any display can be recorded and processed? These emergent, conscious properties of the now fully integrated human-machine dyad are hard to specify. What is not hard to envisage is that they will almost certainly be very different from the nature of consciousness we presently experience.

I cannot leave an envisioned future without at least making one final observation; and this is on the future nature of work (Hancock, 1997). The last century or so has seen a change in emphasis on what composes the content of work. It's not that cognition was not important for example, in nineteenth century work; it was. It is not that the physical requirements of work in our society have disappeared; they have not. But rather, it is the predominance of one over the other as well as the capacity to replace either with an artificial surrogate that have framed our immediate past as to the work experience (Hancock, 2014). But now we have to ask a more fundamental question: what exactly is “work” and what form will it take in the near and more distant future? Heavily influenced by culture and history, work has most often been conceived as something testing, difficult, and often aversive. Work has been a burden to be borne, hard labor to be accomplished, and an arduous necessity for existence. Leisure is often framed as work's antithesis; something desired to be done in hours not devoted to “work.” I have argued before, and argue here, that this is a very limited and impoverished way in which to think of these types of human activity (Hancock, 1997). Thus, I see neuroergonomics as a critical catalyst in a forthcoming “sea-change” in our conception of “work.” In fact, I see a brisance in the wall that separates work from leisure for almost all people. The words obligation, vocation, avocation, and vacation may begin to splinter. Thus, I think neuroergonomics itself will change from the “brain at work” to the activities of the “elaborated person.” In this, I see the title of the recent text by Ayaz and Dehais (2019) not simply as appropriate, but rather as prescient and even prophetic. I take it as self-evident that the progress which will be accomplished in the coming 100 years will dwarf all of those of the whole of human existence. If we are able to avoid the incipient onset of “civicide,” which faces us (Hancock, 2019), what is understood to be “human” at the end of the twenty-first century may well-deserve even a different nomenclature than the species “*homo sapiens*,” that entered it.

## Some Brief Concluding Observations

We have understood the explicit values and operations of cybernetic control loops now for well over half a century (Wiener, 1950; Hancock, 2018b). Enacting human-machine control via adaptive systems employing physiological indicators is itself now more than a third of a century old (Hancock et al., 1985). The employment of such signals, and especially those derived from advancing neuroscience techniques, to the wider swath of technological work is itself now even decades-old (see e.g., Donchin et al., 2000; Parasuraman and Rizzo, 2008). Each of these respective steps is one along a path to ever greater human-machine intimacy. They each, sequentially, attack the barriers of communication between an external system and the operator's internal state. Further, they provide models, simulations, and designs which ought to allow us to consciously and mindfully plan this advancing interaction. But in the end, we must ask an age old question. Because we *can* achieve this ever-increasing degree of intimacy, *should* we necessarily do so? It is a question that pervades all of technology (Hancock, 2018a; Hancock et al., 2019b) and clearly is not confined to the neuroergonomic realm alone. These were some of the wider concerns that equally tasked Raja Parasuraman during his scientific career. His legacy may lead us to our solutions.

Given the contributions of Raja's scientific oeuvre and his continuing concerns as to how technology was used in society, the final conclusion I derive here is a rather simple appeal. It is directed primarily at bench scientists and implores that even as we labor at the rock-face of progress, some portion of our time, effort, and cognition must be given over to the wider ramifications of each experimental procedure that we undertake. This appeal must also be directed to an informed public, whose concern similarly must be for the advances in these overall areas of science and exactly how they will impact and frame future reality. It is so easy for both of these constituencies (working scientists and informed public) to dismiss these moral and ethical conundra with facile utterances such as “not my job,” “not my responsibility,” “not my concern,” or “not relevant at my pay grade,” but it is this indifference to accountability, and its lack of associated moral obligations that can lead us away from the cybernetic dysfunctionality we are obviously experiencing at the present time (Hancock, 2018b). At least in Raja's name, the appeal has now been made here.

## Author Contributions

The author confirms being the sole contributor of this work and has approved it for publication.

### Conflict of Interest Statement

The author declares that the research was conducted in the absence of any commercial or financial relationships that could be construed as a potential conflict of interest.

## Footnotes

^1^Clearly, I do not wish to convey that these are the only antecedents of the mind-brain-machine interface conception which has origins in multiple disciplines ranging from computer science and engineering through cybernetics and all of the neurosciences.
